# Psychosocial risks factors among victim support workers during the COVID-19 pandemic: a study with the Copenhagen Psychosocial Questionnaire

**DOI:** 10.1186/s40359-022-00825-5

**Published:** 2022-05-03

**Authors:** Sónia Caridade, Ana Oliveira, Rosa Saavedra, Rita Ribeiro, Manuela Santos, Iris Almeida, Cristina Soeiro

**Affiliations:** 1 Psychology Research Center, School of Psychology, University of Minho, Campus de Gualtar, 4710-057 Braga, Portugal; 2 Interdisciplinary Center for Gender Studies (CIEG) of the Higher Institute of Social and Political Sciences of the University of Lisbon (ISCSP-UL), 1300-663, Lisboa, Portugal; 3 Portuguese Association for Victim Support (APAV) - PT, Rua José Estêvão, 135 A, Piso 1, 1150-201 Lisboa, Portugal; 4 Interdisciplinary Research Centre on Crime, Justice and Security (CJS) of School of Criminology of Faculty of Law of Porto (FDUP-UP), 4050-123, Porto, Portugal; 5 Center for Research in Neuropsychology and Cognitive Behavioral Intervention, the Faculty of Psychology and Education Sciences at the University of Coimbra, 3000-115 Coimbra, Portugal; 6 Multidisciplinary Research Center of Egas Moniz (CiiEM), Laboratory of Psychology (LabPSI), Egas Moniz Higher Institute of Health Science, Campus Universitário, Quinta da Granja, Monte de Caparica, 2829-511 Almada, Portugal; 7 Institute of Judicial Police and Criminal Sciences, Rua Francisco José Purificação Chaves, 8, 2670-542 Loures, Portugal

**Keywords:** Psychosocial risk (PSR), Occupational health and safety, Victim support workers (VSW), Coronavirus disease (COVID-19) pandemic

## Abstract

**Background:**

Being a victim support worker (VSW) involves exposure to victims' suffering, pain, and traumatic events, which may trigger the risk of VSWs developing mental health problems. Psychosocial risks (PSR) and work-related stress are considered the most challenging issues in occupational safety and health, considering they impact individuals, organizations, and economies.

**Methods:**

The purpose of the present study was to identify the PSR in a sample of 196 Portuguese victim support workers (VSW) (Mean age = 36.49; *SD* = 10.52). A questionnaire with socio-demographic characteristics, variables related to VSW's job, and the Portuguese medium version of the Copenhagen Psychosocial Questionnaire II (COPSOQ II) were used to assess these professionals' perception of PSR factors.

**Results:**

The results reveal that although VSW recognizes some psychosocial factors favourable to their health and well-being, they also identify some PSR that place them at intermediate and severe risk, i.e., emotional and cognitive demands, which are the main areas of risk to the VSW. VSW over 38 years old scored higher in job insecurity, burnout, and offensive behaviours.

**Conclusions:**

These findings give important insights into the areas that must be enhanced in this context involving VSW. Additionally, the results highlight the relevance of encouraging a healthy and supportive work environment, preventing and promoting the health and well-being of VSW, particularly when considering the coronavirus disease (COVID-19) pandemic.

## Introduction

Living conditions and the world of work have undergone significant changes over time. Socio-economic changes increased uncertainty, professional instability, corporate restructuring, increased workload, and pace of work are associated with increased psychosocial risks (PSR). There is a growing lack of boundaries between work and leisure and greater difficulty in balancing personal, family, and professional life [1]. PSR has thus been recognized as a public health concern and one of the greatest challenges for occupational safety and health [2]. According to the European Agency for Safety and Health at Work [3], work-connected PSR involves "all aspects relating to work performance, as well as organization and management and their social and environmental contexts, which have the potential to cause physical, social or psychological harm".

With the outbreak of the COronaVIrus Disease (COVID-19) pandemic, many people have been forced to remote work and face a high workload [4]. An example of this is the health sector or even workers providing support to victims. Remote work may entail some challenges and demands, namely that it implies being more exposed to certain specific risks, such as isolation, difficulties in separating work from domestic tasks, greater risk of conflicts, and domestic violence. In crisis situations such as the one generated by COVID-19, job insecurity is additionally considered, namely the fear of losing jobs, suffering cuts and salary reduction, possible dismissal, and reduction of benefits. Indeed, the research shows the existence of other risks (e.g., greater job instability and financial concerns associated with the pandemic situation) to people's mental health, making them more vulnerable to the development of anxiety and depression problems [5]. It is, therefore, keeping victim support services active, respecting the public security measures imposed by the pandemic situation (e.g., wearing masks and social distancing, rules of self-isolation, and closure of non-essential). In the debate over the indirect effects of the COVID-19 pandemic crisis, it is speculated that the measures imposed to contain the spread of COVID-19 introduced considerable changes in our daily lives [cf. 4, 5, 7] . This may have profound implications for work organization, progressively stimulating greater digitization of work [4], resulting in more layoffs.

### Psychosocial risks in victim support workers

Victim support workers (VSW) are professionals with specific knowledge and training to support victims of crime and violence. In their daily work, VSW listen to traumatic stories and must deal with critical situations daily. Working with victims and offenders poses numerous challenges for the VSW, implying some exposure to experiences of both victimization and offenses, having a potentially negative impact on the emotional well-being of these professionals [7]. There has been an increasing awareness that professionals who support traumatised people are also at risk of developing various psychological problems when considering the specific demands of the tasks involved in their job [8, 9]. The contact or exposure to victims with a traumatic experience, e.g., adult victims with experiences of sexual victimization or sexually abused children, may trigger vicarious trauma in VSW. This is explained by the degree of exposure of the VSW to images of terror, which are considered cruel and emotionally disturbing, and which can promote the occurrence of negative changes in the coaches' cognitive scheme around truth, security, power, independence, self-esteem, and intimacy [10]. Lisa et al. [10] also considered the greater vulnerability of these professionals to the development of burnout. Posttraumatic stress disorder (PTSD) involves different symptoms such as anger, anxiety, depression, intrusive images related to the victims' experiences, flashbacks, intrusive thoughts, difficulties in falling asleep, nightmares, somatic complaints, or physiological activation, which are also presented by VSW [8, 11]. Likewise, Andersen et al. [8] also show that the work-related threats also increased the risk for PTSD in the long term, especially for workers dealing with the forensic population. Similarly, Zammitti et al. [12] show that fear may also play an important role in developing PTSD symptoms during the coronavirus pandemic, particularly in individuals who have experienced negative feelings. However, a meta-synthesis developed by Cohen and Collens [13], based on the analysis of twenty qualitative studies carried out on the experiences of trauma workers, concluded both for the negative emotional potential and the impact of traumatic work, usually presented within the framework of vicarious trauma, but also by growth, as a consequence of workers' involvement in traumatic work. Vicarious trauma, i.e., personal transformations experienced by trauma workers [11], and vicarious posttraumatic growth, i.e., overcoming and positive psychological change experienced [14, 15], thus emerged as two processes that result from an empathic involvement with traumatized victims and occur due to challenges to current cognitive schemas that allow their adaptation [13]. It should be noted that the impact of traumatic victimization tends, however, to differ from one VSW to another, as these reactions are often associated with a complex interaction between organizational issues and the individual worker's characteristics [11].

Exposure to PSR factors in the work and surrounding social environment affects the productivity of organizations, leading to many effects for workers, such as work overload, role ambiguity, lack of social support, or work-family conflict [2]. The development of mental and health disorders, which can involve sleep disorders, anxiety, depression, work accidents, absenteeism, and occupational diseases have been identified as other effects on workers [14]. Side effects at the organization's resilience can be observed, for example, through chronic situations of absenteeism, high turnover, and lack of organizational commitment [2].

The negative impact of stress at work and PSR on physical and psychological health has been the subject of numerous studies [e.g., 14–16], and different instruments are also emerging to identify and assess this phenomenon [17]. The Copenhagen Psychosocial Questionnaire (COPSOQ) is an example of the most widely used instruments to assess PSR and has been used in various occupational sectors [20]. A systematic review and meta-analysis conducted by Molen et al. [19] concluded by moderate-quality evidence that effort-reward imbalance, low procedural and relational justice, high work demand, low support from colleagues and supervisors, high emotional demand, and low decision authority would increase the incidence of stress-related disorders (20% to 90%). In a systematic literature review, Mccormack et al. [16] also found that workload and work settings are the most common work demands and factors that contribute to burnout among applied psychologists.

Three broad categories of PSR are thus identified [2, 20]: (i) job content, such as conflicting demands, lack of role clarity, lack of training and development opportunities; (ii) work organization involving excessive workloads and work intensity, few rest breaks, shift schedules; contradictory superior requirements, ineffective communication, and poor work-life balance, and (iii) work-related interpersonal relationships, e.g., the lack of clarity in the definition of roles, conflicts and poor relationship between workers, lack of opportunities for promotion and development, lack of rewards, job insecurity.

The PSR is one of the greatest occupational health and safety challenges, evidenced by the mental and social demands that certain psychosocial factors (e.g., work organization, working hours, social relationships, work content, and workload) impose on workers. The psychological and social aspects of work are also other factors with a decisive role in the workplace and a considerable and growing impact on the health and well-being of workers [18].

Additionally, the costs of occupational health problems to both productivity and/or health have been recognized [21]. The lack of psychological health at work and the considerable human costs end up having an impact on organizations, society, and the economy. In this sense, it is estimated that the loss of productivity can represent a cost of up to 3.2 billion euros per year for companies worldwide [1]. The greater awareness of the impact that PSR can have on workers means that the concern for the well-being of workers in the workplace has come to assume more and more relevance.

### Present study

Overall, the literature is consensual about the impact that PSR may have on workers' well-being and mental health. This stimulates increased investment research in this field to prevent risk and promote healthy environments for workers, thus resulting in productivity and organizational growth [22]. The Portuguese research focused on PSR factors is scarce. The few existing studies are focused on the workers' health [e.g., 21, 22], active adults, i.e., the general population, COVID-19 professionals involved, or COVID-19 affected professionals, including psychologists [6]. Many of them aimed only to analyse the psychometric characteristics of the COPSOQ [e.g., 23, 24].

There are no known studies on the PSR of VSW in the Portuguese context, so this study is considered innovative in this perspective. It is also the first Portuguese study intending to characterize the work-related PSR in a sample of Portuguese VSW, with a special focus on the COVID-19 pandemic period. Specifically, it is intended to:

(i) characterize the work condition and general support provided by VSW during the COVID-19 pandemic period;
(ii) identify the levels of PSR factors for the health of VSW in each of the Copenhagen Psychosocial Questionnaire II (COPSOQ II) during the pandemic period;
(iii) analyse the PSR factors according to age and working conditions (f2f vs. remote work vs. mixed work) of the VSWs, during the COVID-19 pandemic period

## Methods

### Participants

A total of 196 Portuguese VSW members of the National Support Network for Victims of Domestic Violence (NSNVDV) participated in this study, mainly women (91.8%), with a mean age of 36.49 (SD = 10.52). This study resulted from the research project "VAWDV in Times of Pandemic, namely, characterization, challenges, and opportunities in RS" [26], having as the only inclusion criteria, the integration of VSW on the NSNVDV. Most participants (99%) have high education, mostly graduated in psychology (56.6%), following 18.4% in law (18.4%) and social service (15.8%). The majority (74%) of the participants claimed to work in a victim support institution, presenting an average of 4.88 (SD = 5.65) years of professional experience. Similarly, 86.2% of the participants claimed to have training as a VSW.

### Instruments

**Fig. 1:**
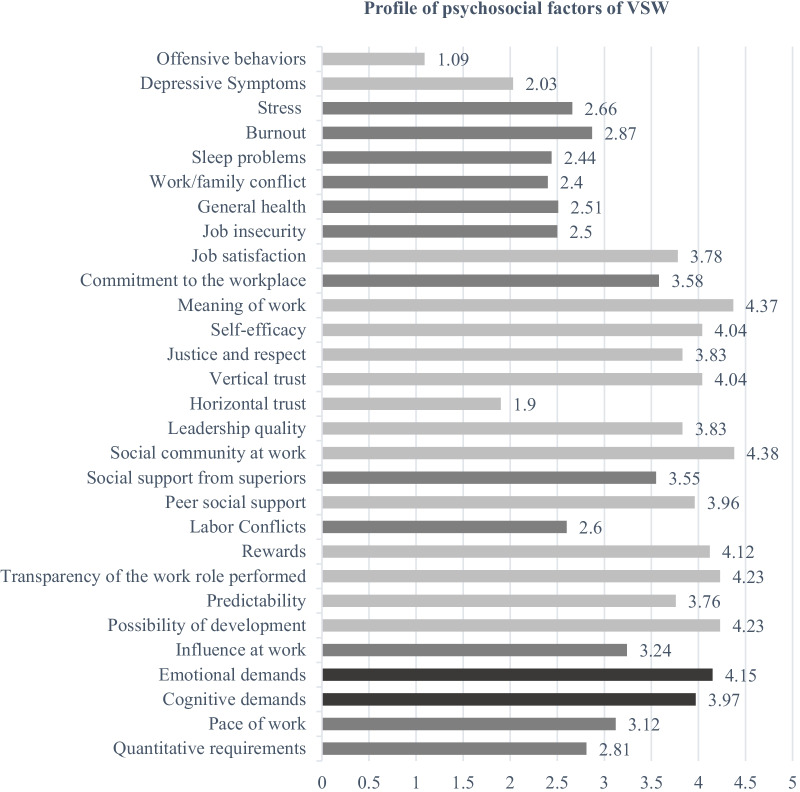
Psychosocial factors profile of VSW. Legend: Light gray: Favourable situation; Dark gray: Intermediate Risk; Black—Health Risk

A questionnaire integrates socio-demographic characteristics (gender, age, level of education, field of knowledge), variables related to VSW job (e.g., type of organization, years of experience), and variables related to work conditions and type of support provided to victims during the lockdown resulting from the generated COVID-19 pandemic, as well as the type of support available before this pandemic situation. As in the original scale, the Portuguese version of the Copenhagen Psychosocial Questionnaire II (COPSOQ II) [25] is available in three versions: short, medium, and long. In the present study, we used the medium version to assess the perception of PSR factors among VSW since it is assumed to be the most suitable for use in occupational health, presenting a complete identification of psychosocial dimensions and not being excessively long. COPSOQ is one of the most widely used instruments, translated into more than 20 languages, for research and PSR prevention in the workplace. It was developed and validated by Kristensen and Borg [27] with the collaboration of the Danish National Institute for Occupational Health in Copenhagen. It is a multidimensional questionnaire that includes numerous dimensions based on an eclectic set of theories on psychosocial factors at work and empirical research [27]. The Portuguese version [25] contains 29 scales (Fig. 1) and 87 items rated on a 5-point Likert scale, ranging from 1 to 5 points. The score of each scale is calculated based on the guidelines' recommendations [25]. These values were then divided using cut-off points based on the Portuguese population, creating three levels with three different colours: low, medium, and high. Low (light gray) means a favourable situation for health, medium (dark gray) a moderate situation, and high (black) a critical risk to health. Most subscales of the medium version of COPSOQ II showed acceptable to good reliability, that is, Cronbach's alpha (α) > 0.7. For 19 of the 29 subscales, Cronbach's alpha was generally above the conventional threshold of 0.70, two scales ranged between 0.60 and 0.70 (vertical trust and horizontal trust), and four scales had reliability of less than 0.60 (rewards, role clarity, social support from colleagues and sense of community at work).

### Procedure

The procedures adopted in this study were the same as those adopted in another study that was part of the aforementioned research project, "Violence Against Women and Domestic Violence (VAWDV) in Times of Pandemic: characterization, challenges and opportunities in distance support” [26]. To carry out this study and to access as many VSW as possible within the NSNVDV, the Commission for Citizenship and Gender Equality was requested to collaborate in the dissemination of the Uniform Resource Locator (URL) with the protocol. First, information about the study (i.e., objectives; inclusion criteria, i.e., only professionals from the NSNVDV in Portugal; data confidentiality and anonymity, and the voluntary nature of the responses) were presented to the participants, followed by the free and informed consent form, which is a mandatory item to be filled in and to proceed with the response to the protocol. The study was carried out after the first wave of the COVID-19 pandemic (between March and May 2020), and the questionnaires were made available through the google forms platform between June and September 2020. The protocol was duly considered and validated by the Ethics Committee [information omitted] in July 2019 (no specific reference attributed), respecting the Declaration of Helsinki. The present work was carried out under funding granted by the Foundation for Science and Technology (FCT).

### Data analysis

The descriptive univariate analyses were computed to characterize the sample, the work condition, and the general support provided by VSW during the COVID-19 pandemic period (objective i). Data analysis was performed and included descriptive statistics using mean (M) and standard deviation (SD) to identify the levels of PSR factors for the health of VSW in each of the COPSOQ II during the COVID-19 pandemic period (objective ii). Bivariate descriptive and inferential statistics were computed to identify the PSR factors of VSW according to age group (independent samples t-tests) and working conditions in the pandemic period (ANOVA) (objective iii). Posthoc tests were performed using the Bonferroni correction to clarify the differences identified by ANOVA. For dimensions measured as multiitem scales, Cronbach's alpha was calculated to assess reliability; an α = 0.70 was deemed acceptable [27]. All statistical analyses were conducted using IBM® SPSS® software, version 27.

## Results

### Work conditions and general support provided by VSW during the COVID-19 pandemic period

During the COVID-19 pandemic, 52.6% of VSW were on remote work functions, 21.9% in mixing work, i.e., remote work and face-to-face (f2f), and 12.8% in f2f work. More than half of the organizations (57.7%) in which the VSW work suspended f2f functions (about 2 months). After the lockdown periods, 48.0% of the organizations returned to normal functioning, and 44.9% implemented a mixed work system, f2f, and remote work.

During the lockdown, VSW have provided support to the victims through telephone more frequently (Mo = Always—43.9%), followed by f2f support (Mo = Sometimes—33.7%) (Table 1).

**Table 1:** Type of support during the COVID-19 pandemic period

Support	Frequency	*f2f*	Telephone	E-mail	Social Applications (e.g., WhatApp)	Video-conference (e.g. Skype)
Frequency (*N*/%)	Always	18 (9.2)	86 (43.9)	25 (12.8)	9 (4.6)	8 (4.1)
	Frequently	53 (27)	73 (37.2)	51 (26)	32 (16.3)	36 (18.4)
	Sometimes	66 (33.7)	23 (11.7)	48 (24.5)	35 (17.9)	25 (12.8)
	Sheldom	36 (18.4)	6 (3.1)	27 (13.8)	37 (18.9)	36 (18.4)
	Never	23 (11.7)	8 (4.1)	45 (23)	82 (41.8)	91 (46.4)
	Mo	Sometimes	Always	Frequently	Never	Never

*N* = Number of cases; *Mo* = Mode

### Psychosocial risks factors experienced by victim support workers: age and work condition

Figure 1 illustrates the distribution of the different psychosocial factors that COPSOQ II allows to extract by the three levels of analysis defined by the respective instrument, i. e., favourable situation, intermediate-risk, and health risk. The psychosocial factors favourable to the health of VSWs (light grey), that is, factors related to values in the workplace, social relationships, personality, and aspects related to offensive behaviour, are more prominent than the other psychosocial factors. The results suggest that the VSW recognize a good working environment, cooperation among colleagues, a sense of community, the meaning of work, the transparency of objectives and responsibilities, and the possibility of learning new things and developing new skills.

At a moderate risk level (dark grey), factors related to job demands (quantity and pace of work), work organization and content (influence on work), social relationships and leadership (conflicts, social support from supervisors), the work-individual interface (commitment to the workplace, job insecurity, work/family conflict) and the health and well-being (general health, sleeping problems, burnout, and stress) are identified (Fig. 1).

Work demands (emotional and cognitive demands) are shown as the only severe PSR factors for the health of VSW (Fig. 1, in black) (Table 2).

**Table 2:** Internal consistency, mean and standard deviation of the COPSOQ II subscales

COPSOQ II dimensions	Subscales (item number)	α	Sample M (DP)	Instrument M (DP)
Demands at work	Quantitative Demands (3)	0.75	2.80 (0.83)	2.48 (0.86)
	Work Pace (1)	*	3.11 (0.97)	3.18 (1.00)
	Cognitive Demands (3)	0.76	3.96 (0.72)	3.79 (0.71)
	Emotional Demands (1)	*	4.15 (0.86)	3.42 (1.15)
Work organization and job contents	Influence at Work (4)	0.74	3.23 (0.85)	2.83 (0.89)
	Development Possibilities (3)	0.71	4.23 (0.61)	3.85 (0.81)
	Meaning of Work (3)	0.84	3.86 (0.80)	4.03 (0.72)
	Commitment to the Workplace (2)	0.90	4.32 (0.76)	3.40 (0.90)
Interpersonal relations and leadership	Predictability (2)	0.84	4.07 (0.83)	3.23 (0.92)
	Rewards (3)	0.30	3.15 (0.58)	3.71(0.87)
	Role Clarity (3)	0.32	3.65 (0.59)	4.19 (0.72)
	Role Conflicts (3)	0.77	3.60 (0.91)	2.94 (0.69)
	Leadership Quality (4)	0.83	4.12 (0.75)	3.49 (0.93)
	Social Support from Supervisor (3)	0.89	3.84 (0.91)	3.13 (0.97)
	Social Support from Colleagues (3)	0.31	3.21 (0.53)	3.44 (0.77)
Work-individual interface	Job Insecurity (1)	*	1.86 (0.67)	3.13 (1.47)
	Job Satisfaction (4)	0.86	4.09 (0.72)	3.37 (0.75)
	Work/Family Conflict (3)	0.76	3.91 (0.73)	2.67 (1.05)
Workplace values	Vertical Trust (3)	0.67	4.27 (0.52)	3.60 (0.60)
	Horizontal Trust (3)	0.65	3.81 (0.78)	2.79 (0.64)
	Justice and Respect (3)	0.73	3.70 (0.69)	3.37 (0.81)
	Sense of Community at Work (3)	0.31	3.00 (0.59)	3.97 (0.81)
Personality	Self-efficacy (2)	0.91	2.39 (1.03)	3.90 (0.67)
Health and well-being	General Health (1)	*	2.42 (1.19)	3.44 (0.91)
	Stress (2)	0.85	2.44 (1.07)	2.70 (0.90)
	Burnout (2)	0.85	2.86 (0.99)	2.70 (0.97)
	Sleep Problems (2)	0.78	2.65 (0.91)	2.46 (1.05)
	Depressive Symptoms (2)	0.79	3.03 (0.90)	2.35 (0.91)
Offensive behaviours	Offensive Behaviours (4)	0.79	1.09 (0.31)	1.23 (0.48)

*M* = Mean; *SD* = Standard Deviation; *α* = Cronbach's alpha

*It is not possible to calculate Cronbach's alpha since the subscale consists of a single item

Analysing the means and standard deviations of the COPSOQ II subscales by age group of VSW, only statistically significant differences were detected in three subscales, as VSW over 36 years old scored higher in terms of job insecurity [*t*(194) = -0.763, *p* = 0.030], burnout [*t*(194) = − 0.998, *p* = 0.030] and offensive behaviours [*t*(194) = − 2.601, *p* < 0.001)] compared to the VSW under 36 years age (Table 3).

**Table 3:** Mean and standard deviation of COPSOQ II subscales by age group of VSW

PSOQ II subscales	< 36 years old M (DP)	≥ 36 years old M (DP)	t (194)
Quantitative demands	2.78 (0.81)	2.83 (0.85)	− 0.442
Work pace	3.07 (0.96)	3.16 (0.98)	− 0.609
Cognitive demands	3.88 (0.75)	4.07 (0.66)	0.220
Emotional demands	4.07 (0.89)	4.24 (0.80))	0.705
Influence at work	3.16 (0.86)	3.32 (0..83)	− 1.326
Development possibilities	4.22 (0.58)	4.24 (0.66)	− 0.229
Meaning of work	3.86 (0.78)	3.87 (0.83)	− 0.114
Commitment to the workplace	4.32 (0.71)	4.34 (0.83)	− 0.280
Predictability	4.18 (0.74)	3.94 (0.92)	2.047
Rewards	3.16 (0.61)	3.15 (0.57)	0.114
Role clarity	3.72 (0.53)	3.56 (0.65)	1.974
Role conflicts	3.75 (0.85)	3.41 (0.95)	2.715
Leadership quality	4.22 (0.67)	4.00 (0.82)	2.041
Social support from supervisor	3.98 (0.87)	3.68 (0.93)	2.276
Social support from colleagues	3.26 (0.49)	3.14 (0.56)	1.528
Job insecurity	1.82 (0.71)	1.90 (0.63)	− 0.763*
Job satisfaction	4.18 (0.72)	3.98 (0.72)	1.867
Work/family conflict	3.97 (0.72)	3.84 (0.74)	1.158
Vertical trust	4.32 (0.48)	4.22 (0.56)	1.419
Horizontal trust	3.86 (0.74)	3.74 (0.82)	1.067
Justice and respect	3.83 (0.64)	3.53 (0.72)	3.022
Sense of community at work	3.09 (0.56)	2.90 (0.60)	2.213
Self-efficacy	2.31 (1.03)	2.50 (1.02)	− 1.317
General health	2.31 (1.19)	2.55 (1.17)	− 1.354
Stress	2.30 (1.02)	2.63 (1.12)	− 2.144
Burnout	2.80 (1.05)	2.94 (0.91)	− 0.998*
Sleep problems	2.66 (0.89)	2.64 (0.93)	0.162
Depressive symptoms	1.94 (0.84)	2.14 (0.97)	− 1.604
Offensive behaviours	1.03 (0.11)	1.15 (0.43)	− 2.601***

***p < 0.001; ** *p* < 0.01; *p < 0.05; *M* = Mean; *SD* = Standard Deviation

Considering the psychosocial factors as a function of the working conditions (f2f vs. remote work vs. mixed work) of the VSW during the COVID-19 pandemic period, statistically significant differences were found at the level of 5 (demands of work, work organization, and job content, personality, health and well-being, offensive behaviours) of the eight dimensions that integrate the COPSOQ II. More specifically, and considering the different subscales of the instrument, there were differences in terms of emotional demands depending on the work condition of VSW [*F*(2, 168) = 5.179; *p* = 0.007]. Post-hoc tests, using the Bonferroni correction, revealed only significant differences between f2f vs. remote work, *p* = 0.005. Significant differences were also found in the development possibilities [*F*(2, 168) = 3.855; *p* = 0.023] and meaning work [*F*(2, 168) = 4.081; *p* = 0.019] and in both subscales. Post-hoc tests show differences only between f2f and remote workgroups (*p* = 0.021; *p* = 0.031, respectively). Also in self-efficacy [*F*(2, 168) = 3.832; *p* = 0.024] differences were found between remote work and mixed groups in terms of the work condition of VSW (*p* = 0.019). Finally, there were significant differences on the stress [*F*(2, 168) = 5.631; *p* = 0.004] and offensive behaviours [*F*(2, 168) = 3.006; *p* = 0.05] subscales, attending on the work condition of VSW. Post-hoc tests, using the Bonferroni correction, revealed significant differences between f2f vs remote work (*p* = 0.010) and f2f vs mixed work (*p* = 0.005) in stress subscale. In offensive behaviours, only significant differences between f2f vs. mixed work were found, *p* = 0.050 (Table 4).

**Table 4:** Mean and standard deviation of COPSOQ II subscales by work condition of VSW during the COVID-19 pandemic period

COPSOQ II subscales	f2f M (DP)	Remote work M (DP)	Mixed M (DP)	F (2,168)
Quantitative demands	2.96 (0.76)	2.88 (0.78)	2.91 (0.84)	*0.105*
Work pace	2.96 (0.76)	2.88 (0.78)	2.91(0.84)	*0.105*
Cognitive demands	3.20 (1.15)	3.13 (0.88)	3.33 (0.97)	*0.674*
Emotional demands	4.36 (0.53)	3.88 (0.72)	4.03 (0.65)	5.179*
Influence at work	4.48 (0.77)	4.15 (0.79)	4.12 (0.79)	2.060
Development possibilities	3.65 (0.60)	3.31 (0.83)	3.01 (0.95)	3.855*
Meaning of work	4.45 (0.50)	4.11 (0.64)	4.33 (0.59)	4.081*
Commitment to the workplace	3.81 (0.71)	3.74 (0.83)	4.05 (0.77)	2.302
Predictability	4.50 (0.61)	4.25 (0.80)	4.36 (0.79)	1.120
Rewards	3.94 (0.81)	4.00 (0.82)	4.13 (0.92)	0.511
Role clarity	3.29 (0.65)	3.15 (0.57)	3.19 (0.59)	0.587
Role conflicts	3.69 (0.59)	3.67 (0.58)	3.65 (0.55)	0.029
Leadership quality	3.63 (0.94)	3.58 (0.89)	3.58 (0.84)	0.030
Social support from supervisor	4.21 (0.68)	4.08 (0.74)	4.16 (0.64)	0.478
Social support from colleagues	3.65 (0.86)	3.80 (0.89)	3.86 (0.79)	0.462
Job insecurity	3.21(0.50)	3.21 (0.47)	3.20 (0.44)	0.011
Job satisfaction	2.08 (0.70)	1.87 (0.65)	1.93 (0.67)	0.981
Work/family conflict	3.87 (0.64)	4.03 (0.71)	4.13 (0.68)	1.146
Vertical trust	3.87 (0.66)	3.89 (0.69)	3.84 (0.70)	0.090
Horizontal trust	4.29 (0.50)	4.23 (0.51)	4.29 (0.58)	0.272
Justice and respect	3.79 (0.82)	3.81 (0.74)	3.82 (0.78)	0.017
Sense of community at work	3.49 (0.69)	3.65 (0.67)	3.75 (0.69)	1.152
Self-efficacy	2.97 (0.54)	3.07 (0.62)	2.78 (0.48)	3.832*
General health	2.74 (1.03)	2.53 (1.05)	2.19 (0.88)	2.774
Stress	3.20 (1.12)	2.42 (1.23)	2.26 (1.07)	5.631***
Burnout	2.58 (1.41)	2.43 (1.04)	2.38 (1.03)	0.267
Sleep problems	3.18 (0.88)	2.96 (1.01)	2.63 (0.96)	2.867
Depressive symptoms	2.92 (0.83)	2.67 (0.91)	2.53 (0.96)	1.423
Offensive behaviours	2.36 (1.04)	2.10 (0.93)	1.89 (0.80)	3.006*

***p < 0.001; ** *p* < 0.01; *p < 0.05 M = Mean; *SD* = Standard Deviation

## Discussion

Considering the potential impact that PSR factors have on the health and well-being of workers and, consequently, on the productivity and growth of organizations, their identification is essential to outline measures that ensure better emotional and time management and stress experienced by workers. This study aimed to contribute to this domain, identifying work-related PSR in a sample of Portuguese VSW, focusing on the period of the COVID-19 pandemic. It should be noted that the identification of PSR constitutes an important opportunity to identify potential risk areas for improvement in the organization of work, that is, a means of identifying and prioritizing problems, to develop and implement appropriate interventions within the workplace [2].

With the outbreak of the COVID-19 pandemic, more than half of the VSW (52.6%) that participated in this study were working from home, thus being more exposed to a set of specific risks, e.g., isolation, confusing boundaries between work and family, greater risk of conflicts and domestic violence, with possible impact on mental health [28]. After the first lockdown, 44.9% of VSW advanced to mixed work, maintaining exposure to the aforementioned risks. Indeed, a Portuguese study developed by Gaspar et al. [6] to analyse the impact of COVID-19 on global health and PSR at work concluded by the greatest PSR at work, namely the bulk of the responsibility, intellectual effort, multitasking, and overall stress. The association of these risks to work tasks, emotional and cognitive demands, health, and well-being enhances the vulnerability of workers and their greater difficulty in managing the personal and professional challenges caused by the COVID-19 pandemic. Furthermore, mental health was affected in the general public when compared to before the pandemic outbreak [31]. A worsening of certain health symptoms (e.g., insomnia, depression, anxiety, burnout, headaches and fatigue), an increase in risk behaviors related to lifestyles (e.g., sleeping habits, physical activity, food, screen time, consumption among others) or changes related to family and work (e.g., associated with confinement and teleworking) were verified with COVID-19 [6]. In the particular case of the VSW, their exposure to victims with traumatic experiences constitutes an increased risk for developing other psychological problems considering the difficulties of their tasks [8, 9], which include vicarious trauma, burnout [10], or even PTSD [11, 12]. On the other hand, the necessary and greater digitization of the work imposed by the pandemic [4] constitutes another challenge for the VSW, requiring specific knowledge and handling of different digital solutions. In this study, VSW reported greater telephone use (43.9% reported using it always and 37.2% using it frequently), opposite to other forms of remote support, e.g., social/mobile applications or videoconference. Such results may be explained by the lack of training of VSW in the use of these technologies and the fact that technological/digital innovations have not been previously considered from a strategical point of view [26]. In fact, the COVID-19 pandemic brought numerous challenges to VSW, namely having to provide remote support, which had never occurred before at such a scale, and there was, until now, a procedural protocol in this regard.

Considering the dimensions and subscales assessed by COPSOQ II, a positive result was found, showing that most psychosocial factors, e.g., self-efficacy, the meaning of work, job satisfaction, and leadership quality, are shown to be favourable to the health of VSW. However, there are also moderate (e.g., work pace, quantitative demands, influence at work, job insecurity, sleep problems, burnout, stress) and severe (cognitive and emotional demands) risk factors that deserve some attention and concern, supporting the development of efforts that enable further management to minimize/eliminate them. Then, this study shows that the most common and severe risk factors for VSW are related to cognitive and emotional demands within the job context, confirming the challenging component of tasks performed by VSW as an area of high risk and challenges [29]. Effectively, it has been shown that VSW, in the specific demands of their tasks of providing support to traumatized populations, are also at risk of developing a variety of psychological problems, including burnout or even PTSD [8, 9].

In the present study, the impact of PSR was uneven, considering the age groups and the working conditions in which the VSW are inserted. Thus, VSW over 36 years old scored more in job insecurity, burnout, and offensive behaviour. These results are contrary to what was observed in the aforementioned Portuguese study developed by Gaspar et al. [6], in which younger professionals, i.e., 35 years or less, present more PSR of work, greater impact of COVID-19 on work intensity, and negative evolution of health symptoms. Considering the characteristics of the sample in the present study, which is mostly comprised of women, it can be hypothesized that this could be a group of women with school-age children requiring greater support during the COVID-19 pandemic period and, consequently, experiencing greater difficulty in balancing job functions and family life [30].

Regarding the working conditions of the VSW, some favourable psychosocial factors were identified. The VSW who were working f2f scored higher in the possibilities of development and meaning of work when compared with those who are performing functions in remote work, which is explained by the vulnerabilities and obstacles inherent in the use of digital tools necessary to contact and support the victims [31], particularly in terms of establishing a relationship and communication with the victim, give emotional support or even for risk assessment purposes. That is why digital solutions should not replace the f2f support but rather constitute an important alternative and complement to assist victims [32]. Also, VSW that were in remote work reported higher scores in self-efficacy when compared to mixed work, which may be understandable due to the greater demands and challenges that the reconciliation of f2f work and remote work imposes on the personal and family life of these specific workers. In PSR, the main differences in the working condition of the VSW were identified at the level of the dimensions of work demands, health and well-being, and offensive behaviours. More specifically, VSW in f2f work scored higher in terms of emotional demands and stress when compared to those were in remote work, differing even in terms of the stress experienced by VSWs in mixed work. Also, in terms of offensive behaviour, VSW from f2f scored higher when compared to those in mixed work. These results on the PSR seem to suggest that the VSW perceive f2f work as representing a greater risk to their health and well-being. The fact that this study was conducted during the first wave of COVID-19 in Portugal, a period in which more measures to contain the disease were being tested, and when there was still great uncertainty about the spread of the virus, may help to understand the greater threat associated with f2f work. Effectively, other authors [12] have shown that fear of COVID-19 fully mediates the relationship between negative affect and well-being and may also partially mediate the relationship between negative affect and PTSD symptoms. This apparent VSW's sense of insecurity regarding f2f work may also be translated into the unpreparedness and inexistence of a plan for organizations to continue to ensure f2f work with the total safety of their workers.

Despite the contributions of this study, it has some limitations that must be overcome in future studies. Firstly, our sample was mainly composed of women (91.8%) because of the greater female representation to perform the role of VSW. When considering that the study was conducted during the COVID-19 pandemic period, it is important to reflect on the results obtained, including, for example, the influence that the adaptations to remote work caused. Subscales such as work/family conflict, support from supervisors, commitment to the workplace, work pace, and quantitative demands in work may reflect some challenges arising from the impact of the COVID-19 pandemic. The instrument used, the COPSOQ II, is not sensitive to this factor, so it will be important in future studies to assess in what conditions and/or contexts the VSW perform their functions, that is, if f2f or remote work. The present study used a quantitative methodology of an exploratory type, using self-report measures, so it is necessary to invest in more qualitative studies that allow an in-depth approach to the phenomenon of PSR involved in VSW through individual interviews or sessions of a focus group. Complementary data collection methods such as observation may also be used. Finally, in the present study, it was not possible to carry out analyses based on the gender of the participants. Considering the evidence that men and women experience different types of trauma or the possibility of gender-specific expressions of emotional distress (e.g., 29), the impact of COVD-19 on gender inequality [33] as well the gender differences in PSR found in the general Portuguese population [6], further studies should thus seek to explore the gender differences involved. Longitudinal designs that allow a deeper understanding of the PSR factors are also necessary in this context.

The findings of this study have important implications for the promotion of the mental health and well-being of VSW and contribute to improving the organization of work and, consequently, increasing productivity and organizational growth. Organisations working with traumatised people must develop effective guidelines and protocols to identify and support workers showing signs of vicarious trauma, i.e., disseminate indicators of vicarious trauma, help procedures, identify formal sources of help, and create appropriate spaces for practice self-care. Other self-care strategies have been suggested, such as taking care of oneself physically and emotionally, getting enough sleep, eating properly, exercising, or having time for self-reflection, being the organizations responsible for promoting it in a safe, supportive, and respectful environment [33]. VSW should be provided with a support network and be able to disclose any concerns. It is also essential to provide training on PSR, health, and well-being in the workplace to promote greater knowledge and awareness of professionals. Therefore, it is important to promote VSW training to make them aware of the risks of their work, how to deal with the challenges of this work, and how to seek support. Implement actions to promote psychological health in the workplace, develop support measures that favour a balance between professional and personal life, and promote peer support strategies, e.g., peer support, are all important strategies that will contribute to minimizing the PSR involved in VSW tasks.

## Conclusions

The present study pursued an important and innovative contribution to the level of research on PSR factors faced by the VSW, considering the COVID-19 pandemic. There are no other studies focusing on this specific theme when it comes to the Portuguese situation. In addition to recognizing some psychosocial factors favourable to VSW health and well-being, the results of this study have allowed us to identify psychosocial factors that place these specific workers at intermediate and severe risk.

The COVID-19 pandemic came to test the resilience and adaptability of all. So, it has also emerged as a potentiating factor for the PSR to which VSW were already subjected. It is, therefore, the time to review the strategic action plans of organizations working in the context of victim support, promoting healthy workplaces, and providing their VSW with resources and skills that will allow them to manage the specific demands of their job and cultivate their well-being and mental health, essential in the necessary resilience of the tasks involved.

## Data Availability

The datasets used and/or analysed during the current study are available from the corresponding author on reasonable request.

## References

[1] Order of Portuguese Psychologists. Código Deontológico da Ordem dos Psicólogos Portugueses [Deontological Code of the Order of Portuguese Psychologists]. 2021. https://www.ordemdospsicologos.pt/pt/cod_deontologico. Accessed on 15 July 2021.

[2] EU-OSHA - European Agency for Safety and Health at Work. Psychosocial risks and stress at work. 2021. https://osha.europa.eu/en/themes/psychosocial-risks-andstress. Accessed on 10 July 2021.

[3] EU-OSHA - European Agency for Safety and Health at Work. Expert forecast on emerging psychosocial risks related to occupational safety and health. 2007. https://osha.europa.eu/en/publications/report-expert-forecast-emerging-psychosocial-risks-related-occupational-safety-and. Accessed on 10 July 2021.

[4] Rigotti T, Yang L-Q, Jiang Z, Newman A, De Cuyper N, Sekiguchi T (2021). Work-related psychosocial risk factors and coping resources during the COVID-19 Crisis. Appl Psychol.

[5] Wilson JM, Lee J, Fitzgerald HN, Oosterhoff B, Sevi B, Shook NJ (2020). Job insecurity and financial concern during the COVID-19 pandemic are associated with worse mental health. J Occup Environ Med.

[6] Gaspar T, Paiva T, Matos MG (2021). Impact of Covid-19 in global health and psychosocial risks at work. J Occup Environ Med.

[7] Zammitti A, Imbrogliera C, Russo A, Zarbo R, Magnano P (2021). The psychological impact of coronavirus pandemic restrictions in Italy. The mediating role of the fear of covid-19 in the relationship between positive and negative affect with positive and negative outcomes. Eur J Investig Health Psychol Educ.

[8] Caridade, S, Sani I. Desafios inerentes à intervenção com vítimas e agressores [Challenges inherent to intervention with victims and offenders]. In: Sani, Ana SC, editors. Violência, agressão e vitimação: práticas para a intervenção. Coimbra: Almedina; 2018. p. 15–32.

[9] Andersen LP, Hogh A, Elklit A, Andersen JH, Biering K (2018). Work-related threats and violence and post-traumatic symptoms in four high-risk occupations: short- and long-term symptoms. Int Arch Occup Environ Health.

[10] Chouliara Z, Hutchison C, Karatzias T (2009). Vicarious traumatisation in practitioners who work with adult survivors of sexual violence and child sexual abuse: literature review and directions for future research. Couns Psychother Res.

[11] Lisa McCann I, Pearlman LA (1990). Vicarious traumatization: A framework for understanding the psychological effects of working with victims. J Trauma Stress.

[12] Steed LG, Downing R. A phenomenological study of vicarious traumatisation amongst psychologists and professional counsellors working in the field of sexual abuse/assault. Australas J Disaster Trauma Stud. 1998;2(2).

[13] Cohen K, Collens P (2013). The impact of trauma work on trauma workers: a metasynthesis on vicarious trauma and vicarious posttraumatic growth. Psychol Trauma Theory Res Pract Policy.

[14] Tedeschi RG, Calhoun LG (2004). Posttraumatic growth: conceptual foundations and empirical evidence. Psychol Inq.

[15] Tedeschi RG, Calhoun LG, Charlotte C (2014). TARGET ARTICLE: “Posttraumatic growth: conceptual foundations and empirical evidence”. Psychol Inq.

[16] Gil-Monte PR, López-Vílchez J, Llorca-Rubio JL, Sánchez PJ (2016). Prevalencia de riesgos psicosociales en personal de la administración de justicia de la comunidad valenciana (España). Lib Rev Peru Psicol..

[17] Mccormack HM, Macintyre TE, Shea DO, Herring MP, Campbell MJ (2018). The prevalence and cause (s) of burnout among applied psychologists: a systematic review. Front Psychol.

[18] van der Molen HF, Nieuwenhuijsen K, Frings-Dresen MHW, de Groene G (2020). Work-related psychosocial risk factors for stress-related mental disorders: an updated systematic review and meta-analysis. BMJ Open.

[19] Suárez-Reyes S, Aguilar-Morales N, Magaña-Medina DE (2020). Instruments to identify psychosocial risk factors at work: a systematic review. J Labor Demogr Econ..

[20] Nübling M, Burr H, Moncada S, Kristensen TS (2014). COPSOQ International Network: co-operation for research and assessment of psychosocial factors at work. Public Health Forum.

[21] Stauder A, Adam S (2017). Quantifying multiple work-related psychosocial risk factors: proposal for a composite indicator based on the COPSOQ II. Int J Behav Med.

[22] Eurofound & EU-OSHA (2014). Psychosocial risks in Europe: prevalence and strategies for prevention title.

[23] Cox T, Griffiths A, Leka S, Gardiner K, Malcolm Harrington J (2005). Work organization and work-related stress. Occupational hygiene.

[24] Muñoz Rojas D, Orellano N, HernándezPalma H (2018). Riesgo psicosocial: tendencias y nuevas orientaciones laborales. PSICOGENTE..

[25] Coutinho H, Queir C (2018). Work-related determinants of psychosocial risk factors among employees in the hospital setting. Work.

[26] Rosário S, Azevedo LF, Fonseca JA, Nienhaus A, Nübling M, da Costa JT (2017). The Portuguese long version of the Copenhagen Psychosocial Questionnaire II (COPSOQ II)—a validation study. J Occup Med Toxicol.

[27] Silva C, Amaral V, Pereira AC, Bem-haja P, Pereira A, Rodrigues V, Cotrim T, Silvério J, Nossa P. Copenhagen Psychosocial Questionnaire: Portugal e Países Africanos de Língua oficial Portuguesa [Copenhagen Psychosocial Questionnaire: Portugal and Portuguese Speaking African Countries]. Aveiro: Departamento de Educação, Universidade de Aveiro.

[28] Caridade M, Saavedra R, Ribeiro R, Oliveira AC, Santos M, Almeida IS (2016). Remote support to victims of violence against women and domestic violence during the COVID-19 pandemic. J Adult Prot.

[29] Kristensen TS, Hannerz H, Høgh A, Borg V (2005). The Copenhagen Psychosocial Questionnaire—a tool for the assessment and improvement of the psychosocial work environment. Scand J Work Environ Health.

[30] Liukkunen U, Halonen T, Liukkunen U (2020). The ILO and transformation of labour law. International labour organization and global social governance.

[31] Vindegaard N, Benros ME (2020). COVID-19 pandemic and mental health consequences: systematic review of the current evidence. Brain Behav Immun.

[32] Caridade S, Sani A. Desafios inerentes à intervenção com vítimas e agressores. In: Sani A, Caridade S, Coords. Coords Violência, agressão e vitimação: práticas para a intervenção. Coimbra: Almedina; 2013. p. 15–32.

[33] Antares Foundatio. Managing stress in humanitarian workers: guidelines for good practice. 2012.

